# Editorial: 90th anniversary of the 1932 Sherrington and Adrian Nobel prize: new insights into initiation and propagation of action potentials and behavioural modulation of reflexes

**DOI:** 10.3389/fncel.2024.1404698

**Published:** 2024-04-03

**Authors:** William Winlow

**Affiliations:** ^1^University of Naples Federico II, Naples, Italy; ^2^University of Liverpool, Liverpool, United Kingdom

**Keywords:** action potential, mathematical reconstruction, quantum phase computation, multiple spike initiation zones, ectopic action potentials, contralateral information exchange

The motivation for the 1932 Nobel Prize to Sir Charles Sherrington and Edgar Adrian was “for their discoveries regarding the functions of neurons”. In the 1890s Charles Sherrington showed how muscular contractions are followed by relaxation and how different reflexes are part of a complicated interplay in which the spinal cord and brain process nerve impulses and turn them into new impulses to muscles and organs (Nobel Prize 1, 1932, p. 1). The prize was awarded jointly to Edgar Adrian who developed methods for measuring electrical signals in the nervous system, and in 1928 he found that these always have a certain size (Nobel Prize 2, 1932, p. 2), i.e. he provided the first experimental evidence for the all-or-none law of nerves and muscles. Here, we stand on the shoulders of these giants of Neuroscience to view current advances in our field of research. This collection of papers consists of three papers on original research, two review articles and one on Hypothesis and Theory.

## Insights into the nature of the action potentials

Over the last few years it has become increasingly clear that action potentials (APs) are rather more complex than was originally suggested by the excellent work of Hodgkin and Huxley in the early 1950s and Rall who provided the cable equation in 1977. Many workers have since shown that mechanical, thermal and optical changes are associated with action potentials (Drukarch and Wilhelmus). Unfortunately, these views are strongly opposed by the neuroscience establishment (Fraser et al., 2018), but these newer findings need to be incorporated into our understanding of APs, which have a multi-biophysical nature. Given all the background information on membrane biophysics it should be possible to mathematically model the propagation of wave ensembles in an individual axon (e.g., Engelbrecht et al., 2015) and several models are available for studying healthy axons (Peets et al.). However, we often only model small parts of the multi-biophysics of cell membranes and do not reconstitute all the components of the AP so only arrive at a partial understanding of its true nature, particularly the mechanical and thermal events that accompany it. For the future, neuroscientists need to consider all the background physical and biophysical details and their interrelationships when modeling action potentials.

At present, the nerve impulse is believed to be a composite of the physiological AP, the AP pulse (a soliton wave) and the computational AP generated by quantum phase processing (Johnson and Winlow) and it is suggested that the brain may function as a quantum phase using phase ternary computation of APs (Johnson and Winlow, 2019). Spatial patterns in the neocortex are formed from individual patterns illuminating the retina and memory are thought to be encoded by reverberatory loops of computational action potentials (CAPs). Similar processes may take place in the cochlea and associated ganglia, as well as ascending information from the spinal cord. At this point it should be noted that the brain functions very differently from artificial intelligence (AI) networks and is not a contemporary Turing machine, as in AI representations, but is frequency modulated phase ternary computation.

## Modulation of reflexes during behavior

Multiple spike initiation zones (SIZs) have been known for some time (e.g., Calabrese and Kennedy, 1974) and may be able to induce different behaviors from different parts of a central neuron. SIZs have now been demonstrated in the mechanosensory touch (T) cells of the medicinal leech (Scherer et al.) which respond to internal synaptic inputs and external factors such as temperature from different SIZs thus providing information to the central nervous system by modulating temporal and rate coding of APs. This leaves a major question as to how this information is processed to provide appropriate behavioral responses in these poikilotherms.

In a sense those neurons exhibiting ectopic action potentials (EAPs) in mammalian brains may also have more than one SIZ. However EAPs have usually been associated with associated pathological conditions, such as epilepsy (Gutnick and Prince, 1972), and originate distally, traveling antidromically toward the cell body but have often been observed in healthy neocortical pyramidal cells in brain slices from the orbitofrontal cortex of mice (Zhang et al.) and are known to occur in the hippocampus. The question is, “Do such EAPs have a role in normal neurophysiological processes?” We can only speculate on their role(s) at this stage, but they can be induced by prolonged orthodromic stimulation of pyramidal cells and may act as a homeostatic block on runaway excitation in both normal and pathological conditions. In other words they may have a behavioral role that is not yet understood.

Previous work has indicated that there is segregation of gamma wave patterns in entorhinal -hippocampal networks (Schomburg et al., 2014). Here (Hernández-Recio et al.) bidirectional information transfer in cortical-hippocampal circuits underlying cognitive and behavioral tasks in anesthetized rats was studied. This work clarifies the ways in which the brain integrates activity from the two sides of the body. They considered bilateral laminar field potentials (FP) across the CA1/dentate gyrus and used correlation and coherence analyses to extract information from five FP generators on each side of the brain. Their results indicated that essentially different streams of activity enter and return to the cortex ipsilaterally, with slow wave activity reflecting increased contralateral information exchange and fast waves associated with ipsilateral processing.

## Conclusion

Information from current studies remains based on the background provided so ably by Sherrington and Adrian, but new concepts of nervous function continuously arrive. Therefore, researchers need to be open to a wide variety of opinion and ideas to further develop our understanding of nervous systems.

## Author contributions

WW: Conceptualization, writing – original draft, writing – review & editing.
